# Overexpression of ß-Arrestin1 in the Rostral Ventrolateral Medulla Downregulates Angiotensin Receptor and Lowers Blood Pressure in Hypertension

**DOI:** 10.3389/fphys.2018.00297

**Published:** 2018-03-28

**Authors:** Jia-Cen Sun, Bing Liu, Ru-Wen Zhang, Pei-Lei Jiao, Xing Tan, Yang-Kai Wang, Wei-Zhong Wang

**Affiliations:** Department of Physiology and Center of Polar Medical Research, Second Military Medical University, Shanghai, China

**Keywords:** β-arrestin1, AT1R, hypertension, RVLM, sympathetic activity, NFκB

## Abstract

**Background:** Hypertension is characterized by sympathetic overactivity, which is associated with an enhancement in angiotensin receptor type I (AT1R) in the rostral ventrolateral medulla (RVLM). β-arrestin1, a canonical scaffold protein, has been suggested to show a negative effect on G protein-coupled receptors via its internalization and desensitization and/or the biased signaling pathway. The major objectives of the present study were to observe the effect of β-arrestin1 overexpression in the RVLM on cardiovascular regulation in spontaneously hypertensive rats (SHR), and further determine the effect of β-arrestin1 on AT1R expression in the RVLM.

**Methods:** The animal model of β-arrestin1 overexpression was induced by bilateral injection of adeno-associated virus containing Arrb1 gene (AAV-Arrb1) into the RVLM of WKY and SHR.

**Results:** β-arrestin1 was expressed on the pre-sympathetic neurons in the RVLM, and its expression in the RVLM was significantly (*P* < 0.05) downregulated by an average of 64% in SHR than WKY. Overexpression of β-arrestin1 in SHR significantly decreased baseline levels of blood pressure and renal sympathetic nerve activity, and attenuated cardiovascular effects induced by RVLM injection of angiotensin II (100 pmol). Furthermore, β-arrestin1 overexpression in the RVLM significantly reduced the expression of AT1R by 65% and NF-κB p65 phosphorylation by 66% in SHR. It was confirmed that β-arrestin1 overexpression in the RVLM led to an enhancement of interaction between β-arrestin1 and IκB-α.

**Conclusion:** Overexpression of β-arrestin1 in the RVLM reduces BP and sympathetic outflow in hypertension, which may be associated with NFκB-mediated AT1R downregulation.

## Introduction

Accumulating evidences have demonstrated that excessive sympathetic outflow is an important contributor to essential hypertension (Schlaich et al., 2004; Hering et al., 2014). The rostral ventrolateral medulla (RVLM) contains pre-sympathetic neurons whose activity is critical for the generation of vasomotor sympathetic tone. The basal activity of pre-sympathetic neurons in the RVLM is a major mechanism responsible for the generation of resting blood pressure (BP) and sympathetic outflow (Zhang et al., 2008; Kumagai et al., 2012). It has been demonstrated that increased angiotensin II (Ang II) mainly acting on angiotensin receptor 1 (AT1R) in the RVLM contributes to high levels of BP and sympathetic outflow (Dampney et al., 2002). It is reported that Ang II signaling in the RVLM is upregulated under hypertensive condition, and blockade of central AT1R is beneficial to cardiovascular dysfunction in hypertension (Muratani et al., 1993; Ito et al., 2003; Dupont and Brouwers, 2010). Accordingly, AT1R has been regarded as an effective therapeutic target for hypertension.

β-arrestin, the one of visual arrestins (β-arrestin1 and β-arrestin2), has been identified as a scaffold protein that mediates G protein-coupled receptors (GPCRs) desensitization and internalization (Oakley et al., 2000; Satoh and Ready, 2005; Zhang et al., 2005). In additional, β-arrestin also triggers G protein-independent signaling pathway (biased pathway) (Lohse and Hoffmann, 2014), usually provoking totally distinct cellular responses (Jean-Charles et al., 2017). It has been reported that β-arrestin mediated AT1R desensitization and its biased signaling pathway may develop beneficial effects for treatment of heart failure and post-myocardial infarction (Lymperopoulos and Bathgate, 2013). For example, mechanical stretch of AT1R in murine heart leads to β-arrestin2-mediated signaling activation, which increases cardiomyocyte survival (Tang et al., 2014). However, little is known the effect of β-arrestin1 and it biased signaling on AT1R. In central nervous system (CNS), β-arrestin2 has been recently proved suppressor effect for hypertension in the RVLM (Wang et al., 2017). Although β-arrestin1 has been demonstrated to cell-autonomously regulate the morphology of microglia and maintain homeostasis of hippocampal neurogenesis (Tao et al., 2015; Li et al., 2016), the role of β-arrestin1 in the central regulation of cardiovascular activity in physiological or pathophysiological conditions still remains unknown. Hereby, the present study is designed to determine the effects of β-arrestin1 in the RVLM on cardiovascular regulation and AT1R expression in hypertension.

First, in this study we observed the expression of β-arrestin1 in the RVLM in normotensive Wistar-Kyoto (WKY) rats and spontaneously hypertensive rat (SHR). Second, we investigated the effect of β-arrestin1 overexpression in the RVLM on cardiovascular function in SHR. Finally, we observed the effect of β-arrestin1 overexpression on AT1R expression in the RVLM.

## Methods

### Experimental protocols

Sixteen-week-old male WKY rats and SHR were purchased from Sino-British SIPPR/BK Laboratory Animal Ltd., (Shanghai China) in these procedures. All experiments were approved by the Animal Care and Use Committee of Second Military Medical University, and were conducted to the principles of the Institutional Animal Care.

### Construction and production of vector-arrb1

The virus packaging was supported by Obio Technology company (Shanghai). The serotype 9 adeno associated virus (AAV9) was used as the vector to carry the rat Arrb1 cDNA (Accession No. NM_012910). The control vector (AAV-GFP) contained all sequence elements except the target gene (Arrb1). The final infectious titer was 3.7 × 10^12^ vector genomes/ml.

### Retrograde labeling of spinally projecting neurons in the RVLM

The rats were anesthetized with chloral hydrate (1.65 ml/kg, ip) and fixed in the stereotaxic instrument. After the dorsal laminectomy, the Fluorogold (FG, 4% in saline, Fluorochrome, LCC, USA) was injected into intermediolateral cell column (IML), as previously described (Stornetta et al., 2001). As previously depicted (Jeske and McKenna, 1992), we chose T2 segment as the optimal locus where FG was injected (50 μm lateral to midline) into IML with 3 sites (800, 900, and 1,000 μm depth to spinal surface) (50 nl/site). After surgery, the rats were treated with lysed penicillin (3,000 units/kg, i.m.) and housed in pathogen-free environment. Three days later, the rats were euthanized and brain stem was harvested for immunofluorescence analysis.

### Injection of AAV into the RVLM

As previously described (Wang Y. K. et al., 2016), rats were anesthetized with continual inhaled isofluorane (3%) and placed in a stereotaxic frame (Shanghai Alcott Biotech). The skull was well exposed and two symmetrical holes (3.0 mm posterior to the lamda point and 2.0 mm lateral to the midline) were drilled upon the dorsal surface of the occipital. AAV particles containing GFP or Arrb1 gene fragment were bilaterally injected (9.5 mm down to the skull surface) into the RVLM by a 32-gauge Hamilton syringe (5 μl). Finally, rats were administrated with lysed penicillin (1,000 units/kg, i.m.) and housed in aseptic conditions. Rats of the experiments were assigned into four groups (WKY+AAV-GFP, WKY+AAV-Arrb1, SHR+AAV-GFP, SHR+AAV-Arrb1).

### Measurement of BP, HR, and RSNA recording

BP and HR (heart rate) in conscious rats were measured by noninvasive tail-cuff system every 4 days after treatments (ALC-NIBP, Shanghai Alcott Biotech) according to our previous study (Zha et al., 2013). Two weeks after overexpression of β-arrestin1, levels of BP, HR, and RSNA were further detected in anesthetized state. In anesthetized rats (urethane 800 mg/kg and α-chloralose 40 mg/kg ip), the right formal artery was catheterized and left renal sympathetic nerve was isolated for recording of BP, HR, and RSNA using a Powerlab system (AD Instruments). The maximum value of RSNA was calculated after rat was euthanized with an overdose of pentobarbital sodium (200 mg/kg), while the baseline RSNA was expressed as a percentage of maximal RNSA after the background noise was subtracted as previously described (Wang et al., 2007, 2014; Peng et al., 2009).

### Acute microinjection of Ang II into the RVLM

As depicted previously (Gao et al., 2008b; Wang et al., 2009, 2014), the anesthetized rats were placed in the stereotaxic instrument, and the dorsal surface of the medulla was exposed. Microinjections into the RVLM (2.0–2.5 mm rostral and 2.0–2.2 mm lateral to calamus scriptorius, 3.0–3.2 mm deep to dorsal surface of medulla) were performed by a multiple-barrel micropipette. A pressor response to L-glutamate (2 nmol) injection was employed to chemically identify the RVLM before the administration of Ang II [100 pmol/100 nl in artificial cerebral spinal fluid (aCSF), Sigma-Aldrich] injection. Changes in BP, HR, and RSNA were continuously recorded by Powerlab system.

### Western blot analysis and PCR

Based on our previous description (Gao et al., 2008b; Peng et al., 2009; Wang Y. K. et al., 2016), Western Blot analysis and PCR were performed for detecting the protein levels of β-arrestin1, AT1R, p-IκBα/ IκBα, and p-p65/p65 expression. After the rats were killed by an overdose of pentobarbital sodium (200 mg/kg, ip), the brain was removed and RVLM tissue was punched on a freezing microtome (−20°C) in reference to rat atlas. The tissue was lysed in cell lysate for resting for 30 min. After centrifugation of the samples, supernatant was left to measure the protein concentration. Since denatured in 100°C water bath, the protein (35 μg) was run on a 10% SDS-PAGE gel and transferred to PVDF membrane (Milipore). Two lanes on both the left and right containing a molecular weight marker, which provides a visual maker of transfer efficiency and marks band position for target protein. The blocked (5% milk in Tris-buffered saline-Tween) membranes were treated with anti-β-arrestin1 (no.32099, abcam), anti-AT1R (no.1173, Santa Cruz), anti-α-tubulin (no.T6074, Sigma-Aldrich), anti-p-IκBα (no.2859, Cell Signaling), anti-IκBα (no.4812, Cell Signaling), anti- p-p65 (no.3033, Cell Signaling), anti-p65 (no.32536, abcam) antibodies overnight at 4°C. After combination with species-specific secondary antibodies containing Strep-Tactin conjugated to horseradish peroxidase (IBA GmbH), the membranes were stained by chemiluminescent agent (Milipore), at last detecting the binds of protein by Syngene Bio Imaging system (Gene Company). The levels of target proteins were normalized to GAPDH or α-tubulin, which served as a loading control.

Total RNA in tissue samples were extracted by using Trizol (Invitrogen, Buenos Aries, Argentina). RT-PCR were performed to obtain cDNA templates by All-in-one cDNA Synthesis Supermix (BIOTOOL, USA) containing the necessary components for reverse transcription. The quantification of target gene expression (AT1a and AT1b) was manipulated using 2x SYBR Green qPCR Master Mix (BIOTOOL, USA) and the amplification reaction was conducted on a CFX Connect real-time PCR System (BIO-RAD, USA) using the following parameters: 95°C for 5 min, 95°C for 15 s, 60°C for 30 s, 72°C for 30 s, step 2,3, and 4 were repeated for 40 cycles followed by 5 s at 65°C. The special oligonucleotides were used for gene detection, and here shows the oligonucleotide sequences as previously described (Bertagnolli et al., 2014; Macchione et al., 2015), AT1a (forward: 5′-AACCCTCTGTTCTACGGC-3′, reverse: 5′-ACCTGTCACTCCAC CTCA-3′); AT1b (forward: 5′-ATT TCA TCG AGA ACA CCA AT-3′, reverse: 5′-TTT GTTAGA CCC AGT CCA AT-3′). All the acquired data was averaged to β-actin mRNA expression levels.

### Immunofluorescence

As depicted in our previous studies (Wang et al., 2014; Wu et al., 2016), after a lethal administration of overdose of pentobarbital sodium (200 mg/kg, ip), the rats were perfused through the aorta with 0.9% saline and 4% paraformaldehyde. Then the brain was removed and fixed in 4% paraformaldehyde (constituted in PBS) for 24 h at 4°C. Thereafter, 4% paraformaldehyde was replaced by 20% sucrose (constituted in PBS) to dehydrate the brain until it sank to the bottom. After swift frozen, the brain was dissected into sections of 20 μm and floated in PBS. The sections were blocked (10% BSA in PBS) for 2 h before incubated with anti-β-arrestin1 antibody (no:32099, abcam) overnight at 4°C. Then Fluorescein Affinipure goat anti-rabbit IgG (H+L) (no.111095, Jackson) was performed as second antibody to mark the sections with red fluorescence. At last, the sections were mounted on slides detected by a laser confocal microscopy (Leica, TCS-SP5).

### Co-immunoprecipitation

Co-immunoprecipitation was performed as previously described (Qin et al., 2014). Protein A/G plus agarose beads (Santa Cruz) first pre-cleared with whole cell lysates, which were then incubated with anti-β-arrestin1 (no: 32097, abcam) under gender agitation overnight at 4°C. Next day the Protein A/G plus was mixed with the lysates and the mixture was incubated for 3 h at 4°C rotating. Following special centrifugation, the precipitation was washed three times with whole cell lysates and boiled in 1x SDS-loading buffer. Finally, the samples were subjected to normal Western Blot procedures.

### Statistical analysis

All of the values are expressed as mean±SE. Differences in β-arrestin1 expression between WKY and SHR were analyzed by unpaired *t*-test. Differences in β-arrestin1 expression among different SHR ages were analyzed by one-way ANOVA with Tukey's multiple comparisons tests. The other comparisons were analyzed by two-way ANOVA with Tukey's multiple comparisons tests. All of the data were analyzed using GraphPad Prism (GraphPad Software, San Diego, CA). *P* < 0.05 was selected as the value to determine statistical significance.

## Results

### Overexpression of β-arrestin1 in the RVLM attenuated BP and basal RSNA of SHRs

**Figure 1:**
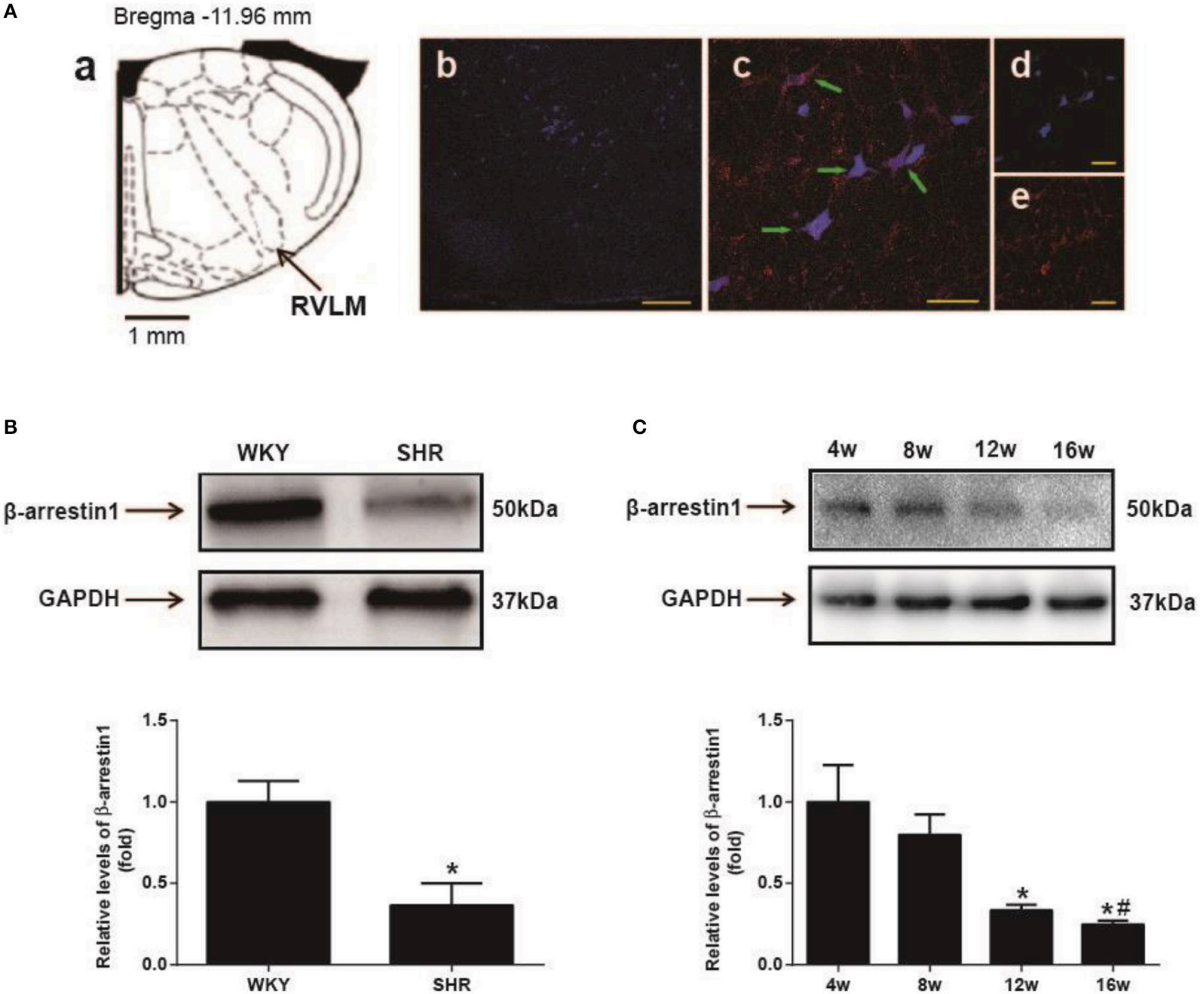
Expression of β-arrestin1 in the RVLM. **(A)** Recording to the standard rat atlas **(a)**, the retrograde tracer FG was restricted in the RVLM **(b)**. **(c–e)** represented the marked neurons (blue color indicating FG immunostaining, and red color indicating β-arrestin1 expression) in the high-magnification and merged image from the area in the RVLM. FG and β-arrestin1 were co-localized in RVLM neurons (green arrows). Scale bars = 200 αm in b and 50 αm in **c–e**. **(B)** Representative gel bands (top) and quantification bar graph (bottom) showed the protein levels of β-arrestin1 in the RVLM of WKY and SHR. *n* = 4 per group, ^*^P < 0.05. **(C)** Representative gel bands (top) and quantification bar graph (bottom) showed the expression of β-arrestin1 in the RVLM of differential ages of SHR. *n* = 5 per group. ^*^P < 0.05 vs. 4 w; ^#^*P* < 0.05 vs. 8 w.

As shown in Figure 1A, β-arrestin1 was expressed in the RVLM neurons labeled by retrograde tracer FG. Furthermore, it was observed that expression level of β-arrestin1 protein in the RVLM was significantly decreased by an average of 64% (*P* < 0.05) in SHR compared with WKY (Figure 1B). In additional, the expression of β-arrestin1 in the RVLM presented a significant decrease at 16th week age compared with 4th week age of SHR (Figure 1C).

**Figure 2:**
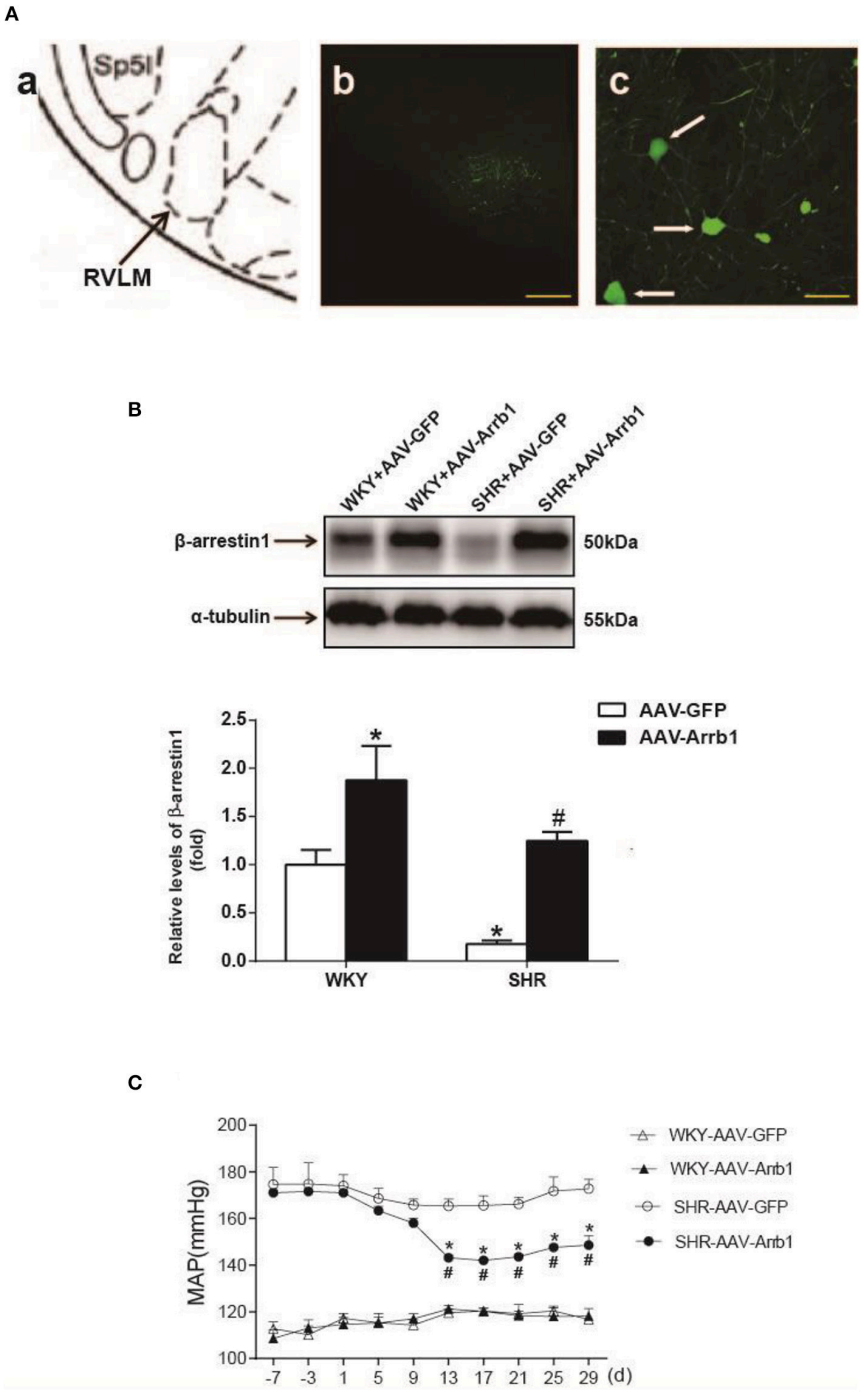
Effects of overexpression of β-arrestin1 in the RVLM on cardiovascular activity in SHR. **(A)** According to the rat's atlas **(a)**, green fluorescent protein (GFP) was expressed in the RVLM area **(b,c)**. Scale bars = 200 μm in b and 50 μm in **c**. **(B)** Representative gel bands (top) and quantification bar graph (bottom) showed the protein levels of β-arrestin1 in the RVLM in the four groups. ^*^P < 0.05 vs. WKY-AAV-GFP group; ^#^*P* < 0.05 vs. SHR-AAV-GFP group. **(C)** Levels of MAP in the conscious groups (WKY+AAV-GFP, WKY+AAV-Arrb1, SHR+AAV-GFP, SHR+AAV-Arrb1). *n* = 5 per group. ^*^P < 0.05 vs. SHR-AAV-GFP group; ^#^*P* < 0.05 vs. d1.

To determine the protective effects of β-arrestin1 in the RVLM in the regulation of cardiovascular activity, β-arrestin1 was overexpressed in the bilateral RVLM using AAV-Arrb1. As shown in Figure 2A, the efficacy of transfection was confirmed by GFP immunofluorescence staining in the RVLM, and the protein levels of β-arrestin1 in the RVLM were significantly upregulated in AAV-Arrb1 groups compared with AAV-GFP groups (Figure 2B). SHR showed an obvious decrease in BP 2 weeks after transfection of AAV-Arrb1 in the RVLM (Figure 2C). Moreover, overexpression of β-arrestin1 in SHR significantly reduced the basal RSNA measured in the anesthetized state (Table 1).

**Table 1:** Baseline levels of MAP, HR, and basal RSNA measured in anesthetized rats in different groups.

	**WKY**		**SHR**	
	**AAV-GFP**	**AAV-Arrb1**	**AAV-GFP**	**AAV-Arrb1**
MAP (mmHg)	107 ± 3.92	108 ± 4.53	168 ± 3.95^*^	138 ± 3.05^#^
HR (bpm)	352 ± 14.2	356 ± 12.7	440 ± 13.8^*^	410 ± 9.3
Basal RSNA (%Max)	16.9 ± 1.54	14.7 ± 0.82	31.7 ± 1.52^*^	22.6 ± 2.73^#^

*Values are presented as mean ± SEM. MAP, mean arterial pressure; HR, heart rate; bpm, beats/min. n = 5 per group*,

* P < 0.05 vs. WKY+AAV-GFP;

# *P < 0.05 vs. SHR+AAV-GFP*.

### Overexpression of β-arrestin1 in the RVLM decreased AT1R expression and NF-κB activation

**Figure 3:**
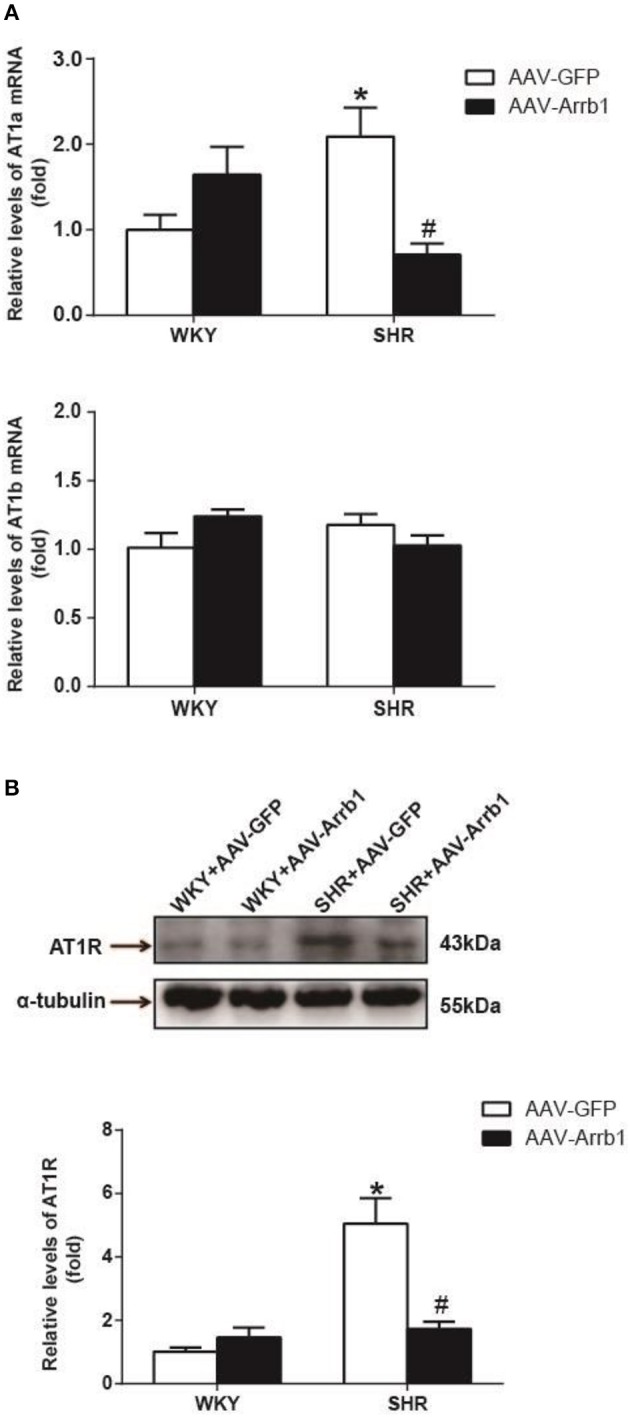
Effects of overexpressed β-arrestin1 on AT1R expression. Relative changes in AT1a/b mRNA **(A)** and protein level [**B**, representative gel bands (top) and quantification results (bottom)] in the RVLM in response to administration of AAV-Arrb1 in SHR vs. AAV-GFP group. No difference was detected in AT1b mRNA expression. *n* = 5 per group. ^*^P < 0.05 vs. WKY-AAV-GFP group; ^#^*P* < 0.05 vs. SHR-AAV-GFP group.

As shown in Figure 3A, the expression of AT1a mRNA was significantly declined by 66%, *P* < 0.05, *n* = 5) after overexpression of β-arrestin1 in the RVLM of SHR, whereas AT1b mRNA was not changed. Similarly, protein level of AT1R was notably decreased by an average of 66% (Figure 3B, *P* < 0.05, *n* = 5) in SHR+AAV-Arrb1 compared with SHR+AAV-GFP.

**Figure 4:**
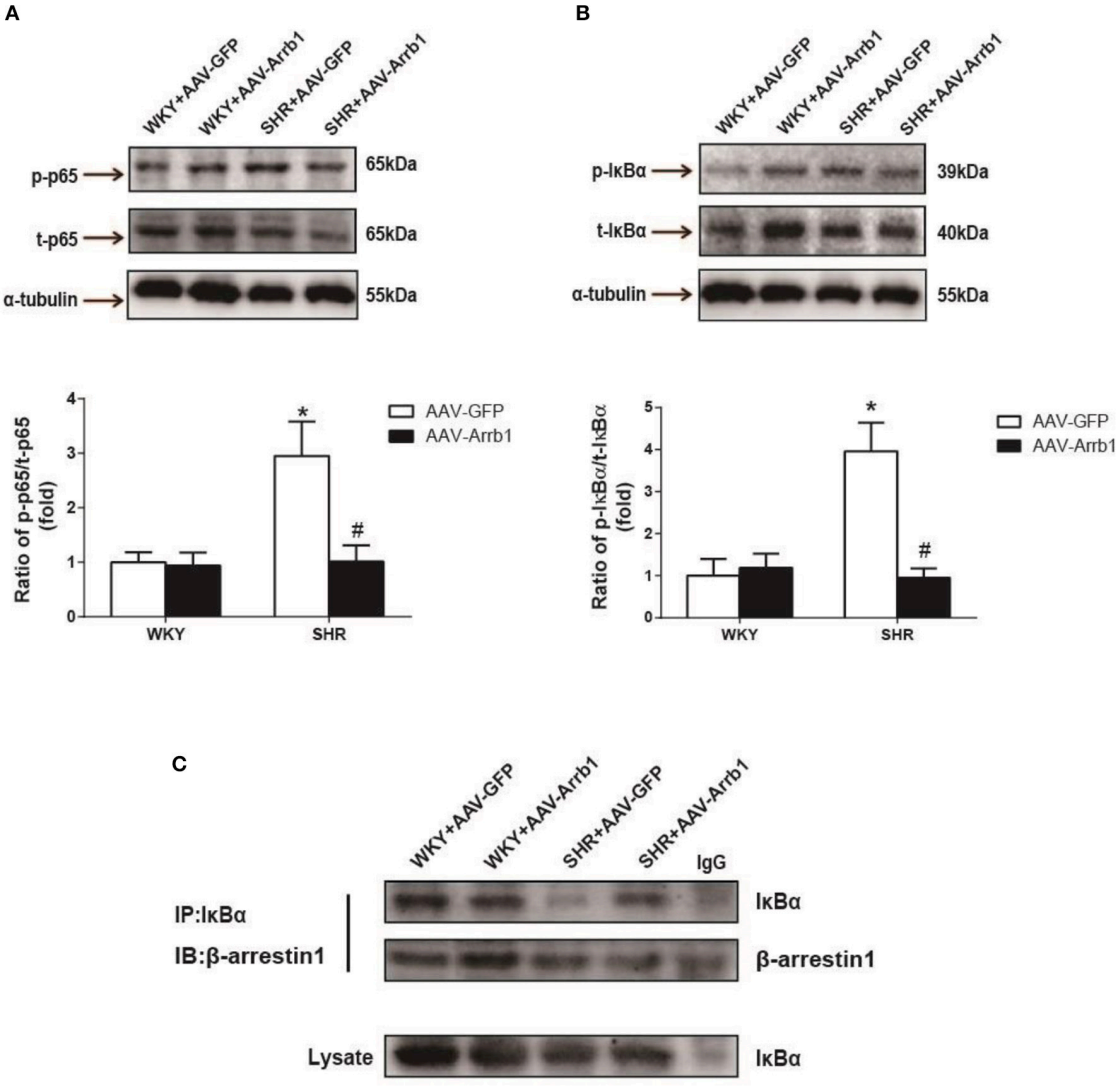
Effects of overexpression of β-arrestin1 on IκBα and NF-κB p65 phosphorylation. Representative gel bands (top) and quantification data (bottom) of phosphorylated NF-κB p65 **(A)** and IκBα **(B)** in response to transfection of AAV-Arrb1 or AAV-GFP in the RVLM. *n* = 5 per group. ^*^P < 0.05 vs. WKY-AAV-GFP group; ^#^*P* < 0.05 vs. SHR-AAV-GFP group. **(C)** Tissues in the RVLM of the four groups were lysed and immuneprecipitated (IP) by anti-IκBα antibody. Western Blot analysis was performed by anti-β-arrestin1 antibody.

It was also observed that overexpression of β-arrestin1 in the RVLM led to a significant decrease in IκB-α (≈76%, *P* < 0.05, *n* = 5) and p65 phosphorylation (≈66%, *P* < 0.05, *n* = 5) in SHR (Figures 4A,B). Furthermore, an augmented interaction between β-arrestin1 and IκB-α in cytoplasm was confirmed after overexpression of β-arrestin1 in the RVLM of SHR by immunoprecipitation (Figure 4C).

### Overexpression of β-arrestin1 in the RVLM blunts the effects of microinjection of Ang II into the RVLM on cardiovascular activity

**Figure 5:**
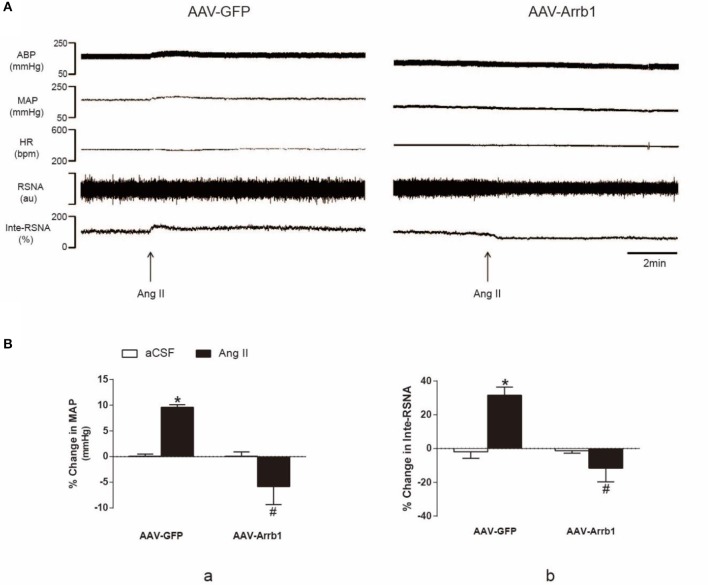
Effects of β-arrestin1 overexpression on Ang II-induced cardiovascular effects in the RVLM. **(A)**, Original tracings of BP, HR, and integrated RSNA (Inte-RSNA) in response to Ang II injection into the RVLM in SHR pretreated with or without β-arrestin1 overexpression. **(B)**, Percent changes in MAP **(a)** and Inte-RSNA **(b)** in response to Ang II injection after β-arrestin1 overexpression. Baseline levels of MAP in four groups before aCSF or Ang II injection are: AAV-GFP+aCSF:161 ± 4.02 mmHg; AAV-arrb1-aCSF:131 ± 4.67 mmHg; AAV-GFP+ Ang II 162 ± 4.26; AAV-arrb1-AngII 131 ± 5.12 mmHg. *n* = 5 per group. ^*^P < 0.05 vs. AAV-GFP+aCSF group; ^#^*P* < 0.05 vs. AAV-GFP+Ang II group.

In order to confirm the effect of β-arrestin1 on AT1R function, Ang II (100 pmol in 100 nl) was microinjected into the RVLM of SHR after treatment with β-arrestin1 or GFP. As shown in Figure 5, the Ang II-induced increase in BP and RSNA (% change in BP: 9.60 ± 0.5 vs. −5.85 ± 3.5%; % change in Inte-RSNA: 31.59 ± 4.8 vs. −11.61 ± 8.1%; *P* < 0.05, *n* = 5) was significantly diminished by the pretreatment with β-arrestin1 overexpression in the RVLM. Interestingly, in some cases, β-arrestin1 overexpression reversed the Ang II-induced cardiovascular excitation.

## Discussion

In the present study, the major findings were observed: (1) β-arrestin1 was expressed in the pre-sympathetic neurons in the RVLM and its expression was significantly decreased in hypertensive rats, (2) overexpression of β-arrestin1 in the RVLM significantly decreased the level of BP and RSNA of SHR, and (3) overexpression of β-arrestin1 in the RVLM decreased AT1R expression and NF-κB activation. Taken together, these findings suggest that β-arrestin1 in the RVLM reduces sympathetic tone and BP in hypertension, which is associated with NF-kB-mediated AT1R downregulation.

β-arrestin1 is ubiquitously expressed in organs and tissues of mammalian animals. In this study, we mainly focused on the pre-sympathetic neurons in the RVLM which exhibit firing patterns to sympathetic nerve activities (Turner et al., 2013). So we firstly identified the expression of β-arrestin1 in the pre-sympathetic neurons in the RVLM using retrograde labeling fluorescent gold. It was found that the expression of β-arrestin1 was significantly reduced in SHR compared with WKY. We further demonstrated its tendency to decrease in the RVLM along with age of SHR. These evidences suggest that decreased β-arrestin1 in the RVLM may be involved in processing of the pathogenesis of hypertension. Enhanced G protein mediated cellular signaling in the central nervous system is a major cause of sympathoexcitation and hypertension (Farnham et al., 2012; Wang H. W. et al., 2016). As a member of β-arrestin family which negatively regulates G protein signaling pathway, the decrease of ß-arrestin1 may be an important mechanism to induce the enhancement of G protein-mediated signaling, thus further leading to hypertension. It has been demonstrated that increased oxidant stress is a common factor for damage of sympathetic vasomotor tone and hypertension formation (Harrison and Gongora, 2009; Chan and Chan, 2012). A recent study has shown that light-induced loss of arrestin levels was prevented or slowed by antioxidants(Wong et al., 2017). Thus, we suggested that the increased excess oxidant stress may contribute to β-arrestin1 downregulation in SHR. Existing evidences have proved the effect of β-arrestin1 on promoting cell survival and protecting ischemic injury as well as its protective role in portal hypertensive gastropathy (Zhu et al., 2013; Tang et al., 2015; Tao et al., 2015). Here in this study, in order to determine if β-arrestin1 in the RVLM is involved in BP control, we constructed the AAV-Arrb1 to promote β-arrestin1 overexpression in the RVLM. As a result, β-arrestin1 overexpression ameliorates hypertension while makes no sense in normotensive state. Therefore, our results indicate that β-arrestin1 in the RVLM is protective for hypertension but may be not involved in maintaining resting BP under normal condition. A recent research has shown that overexpression of β-arrestin2, another subtype of β-arrestin, in the RVLM also improved hypertension by enhancing the cannabinoid type1 (CB1) receptor desensitization (Wang et al., 2017). Taken together, our study indicates that β-arrestins may be beneficial for cardioactivity regulation in the RVLM through different mechanisms.

β-arrestin1 has long been identified as the protein that regulates GPCRs desensitization, internalization, trafficking and signaling (Ferguson, 2001; Gurevich and Gurevich, 2013). As a GPCR, AT1R is intimately related to β-arrestin1 function where previous researches mainly focused on the AT1R-mediated β-arrestin1 recruitment and its signaling pathway (Valero et al., 2016; Young et al., 2017). However, little is known whether β-arrestin1 directly regulates AT1R expression. In the present study, we demonstrated that β-arrestin1 overexpression in the RVLM of SHR apparently downregulated the expression of AT1R protein and AT1a mRNA, indicating that β-arrestin1 may be associated with regulating the AT1R gene transcription. According to our present data, β-arrestin1 didn't significantly upregulate AT1R expression under physiological condition. But it may show a potential tendency to upregulate AT1R by β-arrestin1 overexpression, so we cannot exclude the possibility of ß-arrestin1's effects on AT1R via the other mechanism under normal condition. Importantly, β-arrestin1 was confirmed to downregulate AT1R expression in SHR, suggesting that upregulation of ß-arrestin1 is a potential strategy to lower AT1R expression and ameliorate sympathetic vasomotor tone in hypertension. It has been reported that NF-κB acts as a significant molecular involved in the regulation of AT1R gene transcription and pathophysiology of hypertension (Mitra et al., 2010; Haack et al., 2013). NF-κB in the RVLM has been reported to be involved in some mechanisms of cardiovascular regulation. In additional to AT1R gene transcription, it has been also reported that activation of NF-κB in the RVLM upregulates NOSII and NOSIII gene expression which underlies the elicited sympathetic vasomotor tone excitation and cardiovascular depression and brain stem death (Chan et al., 2004; Tsai et al., 2014). Herein, we hypothesized that β-arrestin1-induced AT1R downregulation was mediated by inhibition of NF-κB activity. Predictably, our results showed that overexpression of β-arrestin1 reduced the phosphorylation of NF-κB as well as IκBα of SHR. In additional, we confirmed that the interaction of β-arrestin1 and IκBα was exactly enhanced in respond to increased β-arrestin1 expression, which is consistent with study previously reported (Witherow et al., 2004). Thus, these data elucidate that the diminished AT1R gene expression results from NF-κB inactivation by combination of β-arrestin1 and IκB-α induced by β-arrestin1. In addition to modulating AT1R expression, we confirm the decreased AT1R function involved in cardiovascular regulation after β-arrestin1 overexpression in the RVLM. Interestingly, we found that overexpression of β-arrestin1 in the RVLM of SHR abolished the cardiovascular excitation in response to microinjection of Ang II into the RVLM. The one possibility is that overexpression of β-arrestin1 elicits the biased signaling pathway, which is the contradictory biological effects of the GPCR signaling (Rakesh et al., 2010; Kim et al., 2014; Abraham et al., 2016). Another possibility is that AT2R function contributes to the Ang II-induced change in BP and RSNA after overexpression of β-arrestin1 in the RVLM. It is reported that AT2R in the RVLM exhibits inhibitory effect on sympathetic outflow (Gao et al., 2008a,b). The effect of RVLM Ang II may be mainly resulted from AT2R activation but not downregulated AT1R by β-arrestin1.

In summary, the present study has shown that overexpression of β-arrestin1 in the RVLM reduces BP and AT1R expression in SHR. Furthermore, the β-arrestin-mediated NF-κB inactivation may be responsible for the decreased AT1R expression in hypertension. Our current data highlight an importance role of β-arrestin1 in central regulation of BP and AT1R in hypertension. It is worth to further determine the possible significance and mechanism of the β-arrestin1-biased signaling pathway in central control of cardiovascular activity in hypertension. The selective central β-arrestin1 biased agonist for AT1R may be a potential strategy for reducing BP and sympathetic outflow in hypertension.

## Author contributions

Study design: J-CS and W-ZW. Performing experiments: J-CS, BL, R-WZ, P-LJ, and XT. Data collection and analysis: J-CS, R-WZ, Y-KW, and W-ZW. Drafting manuscript: J-CS. Revising manuscript content: Y-KW and W-ZW. Approving final version of manuscript: J-CS, BL, P-LJ, XT, Y-KW, and W-ZW.

### Conflict of interest statement

The authors declare that the research was conducted in the absence of any commercial or financial relationships that could be construed as a potential conflict of interest.
